# Emerging Roles of Toxin-Antitoxin Modules in Bacterial Pathogenesis

**DOI:** 10.3390/molecules21060790

**Published:** 2016-06-17

**Authors:** Barbara Kędzierska, Finbarr Hayes

**Affiliations:** 1 Department of Molecular Biology, University of Gdańsk, Wita Stwosza 59, Gdańsk 80-308, Poland; 2 Faculty of Life Sciences, The University of Manchester, Manchester M13 9PL, UK

**Keywords:** toxin-antitoxin complexes, biofilm formation, persistence, pathogenesis, virulence, antibiotic resistance

## Abstract

Toxin-antitoxin (TA) cassettes are encoded widely by bacteria. The modules typically comprise a protein toxin and protein or RNA antitoxin that sequesters the toxin factor. Toxin activation in response to environmental cues or other stresses promotes a dampening of metabolism, most notably protein translation, which permits survival until conditions improve. Emerging evidence also implicates TAs in bacterial pathogenicity. Bacterial persistence involves entry into a transient semi-dormant state in which cells survive unfavorable conditions including killing by antibiotics, which is a significant clinical problem. TA complexes play a fundamental role in inducing persistence by downregulating cellular metabolism. Bacterial biofilms are important in numerous chronic inflammatory and infectious diseases and cause serious therapeutic problems due to their multidrug tolerance and resistance to host immune system actions. Multiple TAs influence biofilm formation through a network of interactions with other factors that mediate biofilm production and maintenance. Moreover, in view of their emerging contributions to bacterial virulence, TAs are potential targets for novel prophylactic and therapeutic approaches that are required urgently in an era of expanding antibiotic resistance. This review summarizes the emerging evidence that implicates TAs in the virulence profiles of a diverse range of key bacterial pathogens that trigger serious human disease.

## 1. Toxin-Antitoxin Complexes

Natural toxins are molecules produced by a wide variety of organisms—plants, animals and microorganisms. These toxins benefit the producing organism by, for example, inhibiting competing species, helping in defense, assisting predation, or promoting infection into a host species. Toxins produced by pathogenic bacteria against their hosts can be classified as either exotoxins or endotoxins. Exotoxins are generated and actively secreted whereas endotoxins are derived from the bacterial outer membrane and are not released until the bacterium dies [1,2]. Bacteria also express highly potent antibiotics, bacteriocins and other compounds that kill or inhibit growth of other, often closely related, microbial species invading their niche [3]. These toxins are not harmful to the microorganisms which produce them. In addition to the preceding toxic factors, most bacterial and archaeal species encode toxin-antitoxin (TA) modules. These systems comprise toxin proteins that disrupt the producing cell’s own molecular processes and cognate antitoxins that block this poisonous activity [4,5,6]. TA modules are distinctive as the toxic proteins are not secreted but instead act only within the producing cell. Thus, TAs are intracellular time bombs which, when activated, can temporarily disable their microbial host from the inside.

**Figure 1:**
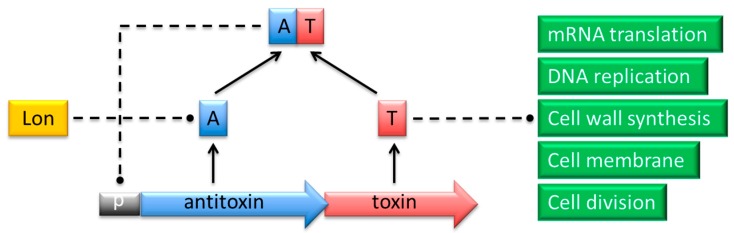
Action of proteins that belong to a typical type II TA complex. The antitoxin (A; blue) and toxin (T; red) genes are co-expressed from a promoter (p; grey) in a single operon. The TA complex negatively regulates transcription from the promoter by binding specific palindromes within the overlapping operator region. In response to certain environmental conditions, the antitoxin is proteolytically cleaved by Lon or Clp proteases (yellow). The toxin is thereby released to act on its specific intracellular target process (green) to induce cell cycle arrest or death.

Typically a pair of toxin and antitoxin genes is encoded within a single operon (Figure 1). The transcription and translation of the genes are tightly coupled to ensure proper stoichiometric quantities of both molecules [6]. The toxin invariably is translated into a protein, whereas the antitoxin functions either as a protein or as a small regulatory RNA. Toxins belonging to different families and exhibiting dissimilar modes of action show structural diversity. Nevertheless, most toxin proteins are comparatively small (<10 kDa) with a compact, globular fold whose core is composed of β-sheets [4,7,8,9]. Protein antitoxins exhibit less obvious common structural patterns. However, numerous protein antitoxins possess regions that lack proper folding which makes them labile to protease digestion [4,7,8]. The general mechanism of TA action relies on differential *in vivo* lifetimes of the two components, which are determined by their biophysical features. Toxin proteins exhibit longer lifetimes compared to the antidotes, which are prone to degradation by cellular nucleases or proteases. When the balance between the two factors is disrupted in response to diverse environmental or intracellular stimuli, the more resistant toxin is activated and reaches its cellular target causing bacteriostatic or bactericidal effects [6,10]. These effects may be advantageous either by dampening cellular activity in response to stress cues, or by inducing the altruistic suicide of a fraction of cells that benefits the population as a whole.

## 2. TA Classes and Function

TA modules are classified into six types based on the mechanism by which the antitoxin inhibits toxin activity [5,11]. In type I cassettes, heteroduplex formation between the antisense RNA antitoxin and the toxin mRNA blocks the translation of the latter [12,13]. The toxin and antitoxin proteins in type II TAs form a stable complex in which the active site of the toxin is hidden by the antitoxin [4] (Figure 1). The antidote in type III complexes is a small RNA that interacts directly with the toxin protein to block its activity [14,15,16]. In type IV systems, a protein antitoxin acts by binding directly to the target of the toxin thereby protecting this target [17]. In contrast, the antitoxin protein functions as a ribonuclease specific against the toxin mRNA in type V TAs [18]. A type VI module has been proposed recently in which the antitoxin acts as a proteolytic adaptor for the ClpXP protease, which is responsible for degradation of the cognate toxin [19,20]. Moreover, bacterial restriction-modification systems and the ppGpp-SpoT metabolite-enzyme pair share certain common features with TA modules and may be considered as TA-like systems in some aspects [11,21]. TAs belonging to types I and II are widely distributed within the prokaryotic world, whereas fewer representatives are known to date for the other classes. However, this situation may change rapidly as extensive bioinformatic analyses together with growing availability of results derived from high-throughput screening methods of microbial genomes such as shotgun cloning lead to the identification of many novel TA families [22].

**Figure 2:**
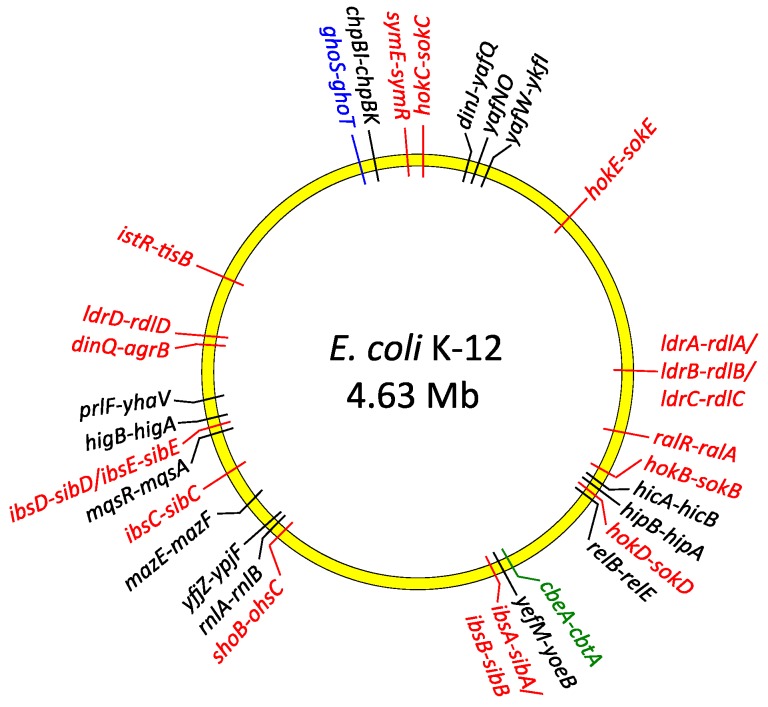
Distribution of type I (red), type II (black), type IV (green) and type V (blue) TA genes on the chromosome of *E. coli* K-12.

TA operons are abundant within the genomes of most bacterial species, often in multiple copies as in *Escherichia coli* (Figure 2), and have no established eukaryotic homologues with comparable functions. TA genes were identified first on plasmids and later on chromosomes. TAs also may be located within other mobile genetic elements including prophages, transposons or superintegrons [7,12,23,24,25,26]. In these locations, TA modules may ensure stable maintenance of the exogenous DNA during cell division. Daughter cells which fail to inherit these elements are eliminated from the bacterial population by the action of the liberated toxin which remains in the cytoplasm of newly-born cells. The antitoxin cannot be replenished in this situation due to the loss of the genetic element that carries the TA genes. TAs encoded on mobile genetic elements play a pivotal role in the maintenance and dissemination among human pathogens of antibiotic resistance and virulence determinants that also are located on these elements [27,28]. On the other hand, growing evidence suggests that the bulk of TA modules primarily act as global metabolic stress managers and are especially beneficial for bacteria living in dynamically changing environmental conditions. Accordingly, comprehensive analyses of microbial genomes have revealed that TA loci are more abundant in free-living prokaryotes than in obligatory intracellular parasites, which are practically devoid of TAs [26]. This observation is in agreement with the hypothesis that TAs help bacteria to cope with diverse external stresses by slowing down cell metabolism, initiating cell cycle arrest or even mediating altruistic programmed cell death [29,30,31]. The role of individual TAs may be to respond to different groups of stresses, although some TAs may have overlapping functions. For example, recent studies of *Pseudomonas putida* isolates from diverse origins suggested that different TA modules are characteristic of clinical strains, whereas other TAs are specific to environmental isolates [32]. Other comparative genomic analyses indicate that epidemic bacterial species may encode significantly more TA modules than non-epidemic species. In this case TAs may act as a defense mechanism that is activated when pathogens are threatened, for example, by the host immune system [33,34]. However, the absence of TA modules in some pathogenic strains and presence in non-pathogenic species of *Rickettsia* does not support this claim [35]. Thus, it is likely that TA modules may possess species-specific functions rather than reflect correlations between bacterial life style and pathogenicity. Nevertheless, growing evidence indicates that numerous TAs directly and actively contribute to pathogenesis of many bacterial species, including promotion of virulence, persister cell production, and biofilm formation. Here we summarize current knowledge of the molecular mechanisms by which TA systems are involved in bacterial pathogenicity.

## 3. The Targets of Toxins are Diverse

The intracellular targets of the toxin factors of TA complexes are diverse. However, numerous type II toxins possess sequence-specific RNA cleavage (endoribonuclease) activity that diminishes protein synthesis and hence cellular metabolism in response to disparate stress and nutritional stimuli. These signals include, for example, amino acid starvation, oxidative stress, and exposure to antibiotics that inhibit transcription or translation. Certain endoribonuclease toxins cleave free mRNA whereas others hydrolyze only ribosome-associated transcripts [4,36].

MazEF is one of the most widespread and well-characterized type II TA complexes (Figure 2). The prototypical MazF toxin of *E. coli* specifically cleaves 5’-ACA-3’ motifs in mRNA transcripts. This cleavage is independent both of the ribosome and of translation and is inhibited by the MazE antitoxin [37,38]. MazF homologues encoded by other species possess sequence specificities that differ both from that of the *E. coli* archetype and from each other. For example, *M. tuberculosis* specifies ten MazF homologues that recognize diverse cleavage motifs in mRNA [39,40]. Moreover, certain MazF toxins also have been shown to cleave both 16S or 23S rRNA and, more recently, tRNA [40]. The cleavage selectivity of MazF homologues and other endoribonuclease toxins may allow targeting of specific pools of RNA whose degradation is advantageous in response to discrete metabolic or environmental stimuli [41].

Like MazF, the type II RelE toxin of *E. coli* (Figure 2) is a sequence-specific endoribonuclease that is counteracted by the cognate RelB antitoxin [42]. MazF and RelE homologues form evolutionarily distinct subgroups of toxins. Unlike MazF, mRNA cleavage by the canonical RelE encoded by *E. coli* is ribosome-dependent, although RelE homologues exhibit differences in this dependency [4,42]. Similar to the MazF family of endoribonucleases, different RelE homologues exhibit varied cleavage specificities [43]. Other toxin families, including the VapC group, also are sequence-specific endoribonucleases [44]. However, not all type II toxins cleave RNA. Instead, certain toxins dampen protein synthesis by inhibiting the translation machinery without mRNA degradation. For example, the HipA toxin inhibits protein synthesis by phosphorylating glutamyl-transfer RNA synthase, whereas the Doc toxin inactivates the essential translation elongation factor EF-Tu by phosphorylation [45,46,47]. Moreover, other type II toxins interfere with the action of topoisomerases that are crucial in maintaining chromosome topology, disrupt cell wall biosynthesis, or interfere with DNA replication [4,19,48,49].

The targets of type I toxins have been less well-investigated than those of type II toxins. The cell membrane generally is the primary target of the former, but type I toxins that mediate DNA or RNA cleavage also have been described [50]. The archetypal type I Hok toxin induces cellular damage by depolarizing the cytoplasmic membrane [51]. The unrelated Fst toxin similarly perturbs membrane integrity, and also impairs chromosome segregation and cell division [52]. In contrast, the type I BsrG toxin does not permeabilize the cell membrane but instead disrupts cell envelope biosynthesis [53]. Although the properties of the ToxIN and homologous complexes are the most well-defined among type III TA complexes, the intracellular target of the ToxN endoribonuclease remains uncertain [54]. The prototypical type IV toxin CbtA binds and interferes with polymerization of the MreB and FtsZ cytoskeletal proteins that are involved in maintenance of cell shape and in cell division, respectively. The CbeA antitoxin does not interact directly with CbtA but instead suppresses toxicity by stabilizing MreB and FtsZ [55]. In the most well-defined type V complex, the GhoT protein toxin disrupts the cell membrane to produce lysed or ‘ghost’ cells. The GhoS antitoxin is a ribonuclease that cleaves the toxin mRNA and disables synthesis of GhoT [18].

## 4. Biofilm Formation is Influenced by the Action of Multiple TA Systems

A biofilm describes populations of microbial cells growing on a wide variety of solid surfaces, either biotic or abiotic, and that exhibit multicellular-like behavior. These cells are enclosed in an extracellular matrix primarily composed of polysaccharides, glycoproteins and DNA, which enables their adhesion and protection from the surrounding environment [56,57]. It is estimated that biofilms are involved in at least 80% of human bacterial chronic inflammatory and infectious diseases [58] and cause serious therapeutic problems due to their multidrug tolerance as well as resistance to host immune system actions [59]. Biofilm development is a dynamic process, which involves the attachment of planktonic cells to the solid surface, microcolony formation followed by cell immobilization, maturation of biofilm architecture into a three dimensional structure, and finally detachment and release of cells back into the planktonic state [57]. The transition between planktonic and biofilm phases requires precise and well-coordinated regulation of expression of many genes at different stages of biofilm development [60]. Cells within biofilms show reduced growth rates, as well as differences in the expression of specific genes compared to suspension cultures [61].

**Figure 3:**
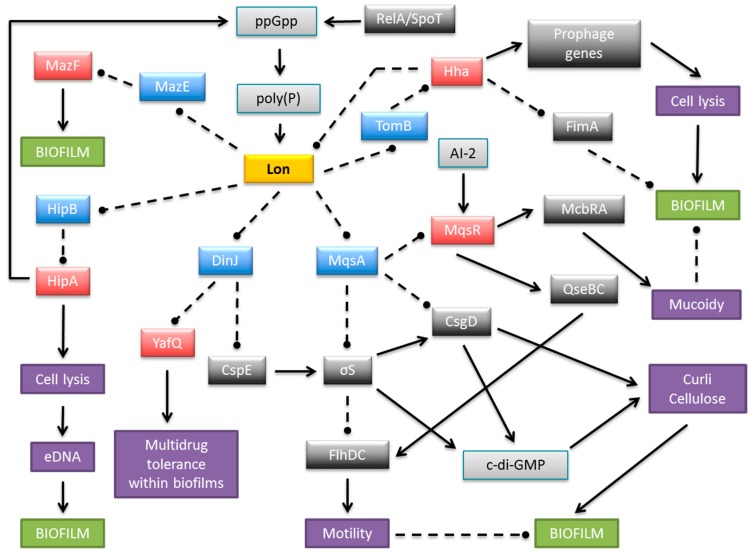
Schematic representation of a complex and multilayered protein network engaged in biofilm development in *E. coli* via TA systems. The central part illustrates Lon protease as an example of antitoxin degrading proteases, which also include ClpXP and ClpAP. Only the relevant, most well-described interactions are displayed. Coloured boxes denote toxins (red), antitoxins (blue), Lon protease (yellow), other proteins (dark grey), small molecules (light grey), and cellular processes and structures (purple). Arrows, stimulation; broken lines ending in balls, inhibition.

Biofilm development is strongly stimulated by environmental conditions and stress genes are involved in biofilm formation [60]. Moreover, biofilm production is influenced by multiple TA systems [57,62,63,64]. The most penetrating insights into the role of TAs in biofilm organization come from research on *E. coli* biofilms. At least 34 TA modules have been identified on the *E. coli* K-12 genome (Figure 2). Cellular proteases, including Lon, ClpXP and ClpPA, seem to be the key players that mediate the environmental stress response by activating multiple TA systems via degradation of the antitoxins (Figure 3). With one known exception, TA modules implicated to date in biofilm formation in *E. coli* belong to type II in which both toxin and antitoxin are proteins. The MqsR-MqsA type II pair was first documented to be engaged in a complex regulatory gene network, which promotes biofilm formation. The antitoxin MqsA directly inhibits the expression of a master regulator of stress, RpoS [65]. This stationary-phase sigma factor controls the expression of up to 10% genes involved in the response to different stresses, such as temperature shock, starvation and growth shift from exponential to stationary phase [66]. MqsA also decreases the production of CsgD, the master regulator for biofilm formation [67] (Figure 3). CsgD transcriptionally activates curli gene expression, as well as the gene for diguanylate cyclase (AdrA) which synthesizes cyclic diguanylate (c-di-GMP) that is implicated in cellulose production [68]. Both curli and cellulose are important components of biofilms. Moreover, the *csgD* gene is transcribed by RNA polymerase which contains the stationary phase sigma subunit, RpoS, which in turn represses the expression of *flhD* which encodes a transcriptional regulator of flagella biogenesis. Thus, in the absence of stress MqsA increases motility by activating *flhD* through *rpoS* as well as *csgD* inhibition (Figure 3). Under stressful conditions, MqsA degradation by Lon protease leads to derepression of *rpoS* and *csgD* and inhibition of *flhD* [65]. Activation of *rpoS* also increases concentrations of the internal messenger c-di-GMP. This regulatory network leads to decreased motility and increased biofilm formation. Therefore, the role of the MqsA antitoxin seems to be the inhibition of biofilm formation in the absence of stress and activation of this mode of growth under unfavorable conditions (Figure 3) [65]. 

The MqsR toxic component of the MqsRA system also participates in the regulation of biofilm production (Figure 3). The expression of *mqsR* is stimulated by the cross-species bacterial communication signal autoinducer 2 (AI-2), which controls chemotaxis, flagellar synthesis, and motility in *E. coli* [60,62,69]. MqsR acts via the motility regulator system QseBC, which transcriptionally induces *flhDC*, the master regulator of flagella and motility genes [69]. In addition, MqsR stimulates expression of the transcription factor McbR [69], which, in turn, inhibits the synthesis of McbA, thereby preventing colanic acid production [70]. Colanic acid is an exopolysaccharide that is important for the formation of three-dimensional architecture of mature biofilms [71,72]. Moreover, the GhoST type V TA pair, which is regulated post transcriptionally by the MqsR toxin, impacts early biofilm formation and swimming motility [18].

The antitoxin DinJ of the YafQ-DinJ TA pair also affects the general stress response by indirectly decreasing RpoS levels. It has been demonstrated that DinJ reduces the expression of RpoS by repressing the *cspE* gene that encodes the cold-shock protein CspE. CspE is an enhancer of translation of *rpoS* mRNA [73]. Moreover, the YafQ toxin mediates tolerance of *E. coli* biofilm cells to multiple, but specific, antibiotics [74].

Further insights into the relation between TA modules and biofilm development are provided by the Hha-TomB (also known as Hha-YbaJ) pair [62,75]. Transcription of both genes is highly induced in biofilms [60]. Hha inhibits biofilm formation by preventing the synthesis of type I fimbriae via transcriptional inhibition of *fimA* and *ihfA* genes as well as by inhibition of their translation via rare tRNAs [75] (Figure 3). Thus, Hha functions as a biofilm inhibitor that promotes dispersal of cells from the mature biofilm, possibly to promote colonization in distant niches. Hha also activates several prophage genes that are involved in lysis of cells, for example the *yfjZ* gene coding for YfjZ toxin of the YpjF-YfjZ TA pair found in a cryptic prophage of *E. coli* K-12 [75,76] (Figure 2). Moreover, the expression of ClpXP and Lon proteases, which are responsible for degradation of many antitoxins, is stimulated by Hha. As a consequence the cognate toxins are liberated to exert their inhibitory effects on bacterial development [75]. Other data have demonstrated that Hha overproduction activates genes encoding the toxins and antitoxins of the YefM-YoeB, DinJ-YafQ and RelBE systems [77]. Moreover, in an *E. coli* strain deleted of five type II TA cassettes (*mazF-mazE, relE-relB, chpB, yoeB-yefM, and yafQ-dinJ*) biofilm development was influenced via control of fimbriae and reduced dispersal through the YjgK (TabA) protein [77]. However, contrasting data were obtained by Kolodkin-Gal and co-workers, showing that only *mazEF* and *dinJ-yafQ* among these five cassettes participated in the promotion of biofilm development through a mechanism related to their role in mediating cell death [78].

The HipBA TA system influences *E. coli* biofilm formation through the production of extracellular DNA (eDNA) [79]. eDNA is an important structural component of the biofilm matrix and directly enhances biofilm adhesion [80,81]. The production of the HipA toxin affects the integrity of *E. coli* cells and may induce the lysis of a subpopulation of cells, which thereby increases eDNA concentrations in biofilms [79]. HipA also is implicated in biofilm formation in the presence of several drugs [82,83]. 

The research summarized above emphasizes TA modules as regulatory elements that play roles in biofilm formation that extend beyond the transcriptional autoregulation of TA loci in *E. coli* (Figure 3). Much less in known about the influence of TA systems on biofilm development in other bacterial species. However, a few examples have been described. *Treponema denticola* is a Gram-negative, spirochete bacterium that is implicated in periodontal disease. *T. denticola* biofilm forming cells have increased expression levels of several putative TA homologues compared to planktonic cells suggesting that these TAs may exert a role in biofilm persistence [84]. Moreover, RelE and VapC toxin homologues in the opportunistic pathogen *Burkholderia cenocepacia* contribute to biofilm formation [85]. Finally, *relBE* systems in *Vibrio cholerae* were shown recently to affect biofilm maturation [86].

The ability to form surface-attached biofilm communities is an important survival strategy for microbial cells. Bacterial biofilms contain an increased prevalence of persister cells, which are insensitive to many different factors, including antibiotics [87]. Biofilms seem to function as protective habitats for persisters, allowing them to escape from the immune system response. Accordingly, Lewis [88] proposed a model explaining the relapse of chronic infections in which antibiotics kill the majority of cells, the immune system eliminates both regular cells and persisters, and the only remaining live cells are persisters embedded within the biofilm. Once the level of antibiotic drops, persisters from the biofilm can revive and the infection relapses [88]. 

## 5. Persistence Emerges in Response to the Activation of Diverse TA Modules

### 5.1. Toxin Activity and Persister Cell Formation in E. coli

Persistence represents a phenotype exhibited by a small fraction of a bacterial population that temporarily enters a dormant state characterized by reduction of growth rates and metabolic activity. Entry into dormancy allows cells to survive unfavorable conditions and to return to the active state when environmental parameters improve. Most antibiotics, as well as many other environmental threats, affect only growing and dividing cells, hence persisters are more protected from killing. It was demonstrated that persisters can arise either stochastically as a result of single cell specific fluctuations in gene expression [89] or in response to a variety of environmental conditions including starvation, oxidative stress, SOS response, carbon source transition and the presence of antibiotics [90,91,92,93].

**Figure 4:**
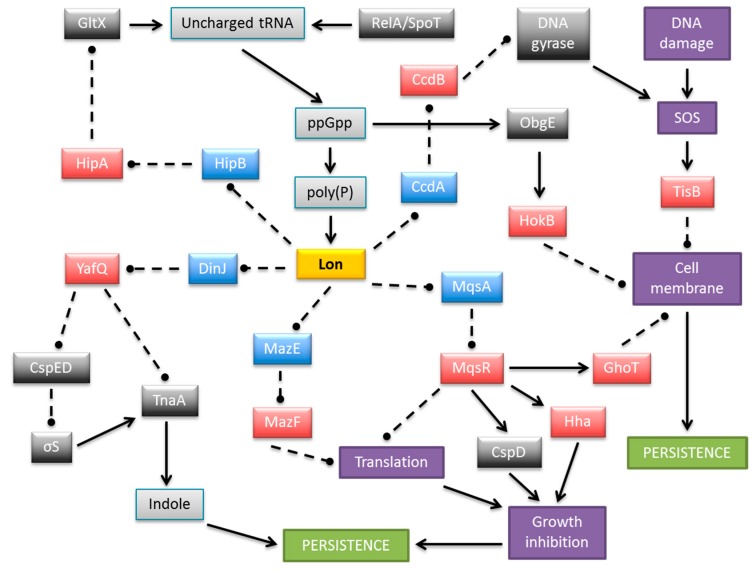
Schematic representation of a complex and multilayered protein network engaged in persistence formation in *E. coli* via TA complexes. The central part illustrates Lon protease as an example of antitoxin degrading proteases, which also include ClpXP and ClpAP. MazEF is shown as an exemplar type II module in which the toxin is an endoribonuclease. Only the relevant, most well-described interactions are displayed. Colored boxes denote toxins (red), antitoxins (blue), Lon protease (yellow), other proteins (dark grey), small molecules (light grey), and cellular processes and structures (purple). Arrows, stimulation; broken lines ending in balls, inhibition.

The contribution of individual TA genes to persistence is difficult to assess because of the functional redundancy of the multiple TA modules encoded within most bacterial chromosomes. However, it has been established unequivocally that TA systems play important roles in persister cell formation [63,64,93,94,95]. Transcriptomic analysis of *E. coli* population fractions enriched for persisters revealed that these cells showed significantly increased levels of TA mRNAs [96,97]. Moreover, persistence can be induced by overproduction of toxins encoded by TA loci and the stimulation of antitoxin gene transcription can reverse this effect [96,97,98,99,100,101]. Accordingly, successive deletion of ten endoribonuclease coding TA systems (RelE, YoeB, HigB, YhaV, YafO, YafQ that cleave mRNA positioned at the ribosomal A site, and MazF, ChpB, MqsR, and HicA that cleave RNA in a site specific manner independent of the ribosome) from the *E. coli* chromosome (Figure 2) produced an additive effect on persister formation, finally reducing persistence more than 100-fold compared to the wild-type strain [90]. At least five TA loci need to be knocked out in *E. coli* before a significant effect on persister frequency was observed [90]. These results illustrated that the formation of at least 99% of persisters in the exponentially growing culture of *E. coli* depends on the TA loci, whereas less than 1% of the persisters arise from random fluctuations in the growth rates of single cells [102]. In support of these results, overexpression of Lon, the protease that degrades most antitoxins, enhanced toxicity and thereby increased the fraction of persisters, whereas the absence of Lon decreased persister formation. The only known activator of Lon protease is inorganic polyphosphate (poly(P)) whose level is influenced by the intracellular concentrations of the stringent response alarmone (p)ppGpp. Thus, (p)ppGpp, which is produced in response to many different types of stresses, induces persister formation by activating TA modules through poly(P) and Lon protease function [103]. A model explaining more specifically the mechanism by which (p)ppGpp and the HipBA type II system mediate persistence was elaborated recently [45,93,104]. Here, free HipA toxin inactivates glutamyl-tRNA synthetase (GltX) by phosphorylation which increases the rate of uncharged tRNA^Glu^ loaded at the A site of the ribosome [45,105]. RelA-dependent (p)ppGpp production is triggered as a result. (p)ppGpp competitively blocks exopolyphosphatase which is the enzyme that degrades poly(P). In turn, poly(P) is synthesized by polyphosphate kinase and activates Lon to degrade type II antitoxins, including HipB. In these conditions the cognate toxin endoribonucleases are liberated causing inhibition of translation and cell growth thereby inducing persistence [45,95,104] (Figure 4).

The rate of global cellular translation is low in the persistent state, which favors a low antitoxin-toxin ratio due to the instability of the former. In this situation, transcriptional autorepression of TA loci is abolished and thereby a high rate of TA transcription and toxin production is promoted which explains the elevated levels of TA mRNAs observed in persisters [96,97]. However, when the Lon-mediated degradation rate of antitoxins returns to a normal level, the rapid production and accumulation of the antitoxins results in quenching of toxin activity and resuscitation of cell growth. This pathway of toxin activation is supported by mathematical modeling [106]. A similar result was obtained by Rotem and co-workers who found that *E. coli* enters a dormant state once the toxin level crosses a threshold, and that the length of the dormancy depends on the toxin’s level [107]. Mathematical modeling of the cooperation between multiple TAs in promoting persistence frequency also has been presented [108].

Nevertheless, the above model is suitable only for persistence mediated by type II TA systems. Moreover, persisters are still formed without (p)ppGpp, although at lower levels. Additionally, the MqsR, MazF, GhoT, and YafQ toxins are able to induce persistence in the absence of (p)ppGpp, poly(P), and Lon [97,102]. Apparently, the persistence model which involves (p)ppGpp, poly(P), and Lon is valid only in a limited number of cases. It has been proposed that the ability of a cell to enter the persister state is inversely related to its growth rate. Thus, cells activate the production of toxins in response to stress as a strategy to reduce growth and to induce persistence in a small subpopulation of cells. Therefore, it appears that persistence does not solely depend on (p)ppGpp but rather on the growth rate of the bacterial culture [109]. Accordingly, very limited overproduction, resulting in minimal inhibition of cell growth, of not only TA toxins but also unrelated, non-TA proteins, such as the DnaJ chaperone of *E. coli* and PmrC inner membrane protein of *Salmonella enterica* subsp. *enterica* serovar Typhimurium (*S. typhimurium*), lead to the production of persister cells tolerant of ampicillin and ciprofloxacin [99]. Therefore, expression of many genes in addition to TA modules can drive the production of dormant cells [99,110,111].

Transcriptional control of TA gene expression plays a vital role in preventing inadvertent toxin activation [6] and potentially in controlling entry into and exit from the persistent state [10]. Antitoxins encoded by type II cassettes are sequence-specific DNA binding proteins that typically recognize their promoter regions poorly, which results in weak, but significant, transcriptional repression of the downstream TA genes. Under these conditions, the levels of antitoxin are sufficient to sequester the toxin molecules. Similarly, when the TA ratio is balanced, toxin interaction with the antitoxin enhances the DNA binding affinity of the latter, which induces strong repression (Figure 1) and maintains an effective TA stoichiometry. However, an increased concentration of toxin, such as occurs during persistence, destabilizes DNA binding by the antitoxin, which results in strong transcriptional de-repression of the TA genes. Sufficient antitoxin is then produced to capture the excess toxin and to re-establish repression. Thus, cells can switch from persistence into a growing phase. The differential transcriptional autoregulation of TA gene expression based on different TA ratios is denoted conditional cooperativity [10]. Although not universal among type II TAs, conditional cooperativity nevertheless appears to be a key strand in achieving bistable toxin concentrations that dictate the transition between persistent and actively growing states [112,113].

### 5.2. Multiple Mechanisms for TA-Induced Persistence

As described above, it is well established that type II endoribonuclease toxins inhibit general cellular protein synthesis, which leads to an increase in persister cell formation. However, it also has been demonstrated that induction of certain endoribonucleases leads to the enrichment or reduction of specific transcripts [63]. Accordingly, production of YafQ toxin increases persister cell formation through a significant reduction of tryptophanase (TnaA) levels caused by the fact that the *tnaA* mRNA has 16 putative YafQ cleavage sites [114]. Furthermore, *tnaA* is activated in the stationary phase by RpoS whereas YafQ reduces *rpoS* expression indirectly by diminishing production of the CspC and CspE cold-shock proteins. Hence, the concomitant reduction in RpoS and TnaA protein levels by YafQ leads to reduced levels of indole which is the product of TnaA activity [114]. Indole is a quorum-sensing signal that influences multiple aspects of bacterial physiology and is an important factor in the transition from growth phase to the stationary phase (Figure 4).

In contrast to the preceding activity of YafQ, the sequence-specific endoribonuclease activity of the MqsR toxin leads to the enrichment of certain transcripts that encode stress associated proteins. Moreover, persister cells produced by antibiotic stress induce MqsR, which influences production of other toxins, Hha and CspD [115,116]. The *ghoT* mRNA also is enriched by MqsR induction. The type V GhoST cassette increases persistence by damaging the cell membrane. The mechanism by which GhoT leads to the loss of membrane integrity and induces a dormant state probably relies on the modulation of proton pump activity or interaction with other membrane proteins. Since the activity of MqsR results in the enrichment of the transmembrane protein GhoT and also inhibits the expression of many genes, including these coding for the OmpA and OmpF outer membrane proteins, it may indicate that MqsR increases persistence through a tight control of membrane permeability [117,118].

The *tisB/istR1* module of *E. coli* (Figure 2) is a type I TA that is involved in the persistence phenotype under conditions of SOS induction [119]. The hydrophobic transmembrane peptide toxin TisB induces persister cell formation by decreasing both the proton motive force and ATP levels upon antibiotic stress, thus leading to the promotion of a dormant state. Similar to GhoT, the activity of TisB leads to damage of the inner membrane. TisB-dependent persisters were observed only in exponential growth when the SOS regulon is maximally expressed upon DNA damage [120]. Interestingly, the SOS response system also activates several other TA genes in *E. coli*, whose promoters contain a motif that may be recognized by the LexA transcriptional repressor: *symER, hokE* and *yafN/yafO* [121].

The type I *hokB* toxin gene induces bacterial multidrug tolerance in a (p)ppGpp-dependent manner, via transcriptional activation by universally conserved GTPase ObgE (also known as CgtA). The HokB peptide provokes a collapse in the bacterial membrane potential. However, moderate levels of HokB cause membrane depolarization, which does not kill cells, but ultimately results in persistence [94,122]. Collectively, the preceding findings show that certain membrane-acting toxin proteins are elements that are actively involved in persistence (Figure 4).

Plasmid encoded TA systems are loci that stabilize these elements in bacteria. However, it was shown that the *ccd* TA operon either located on a plasmid or on the *E. coli* chromosome, also plays an indirect but significant role in the formation of persisters. Stress conditions activate Lon protease, which causes CcdA antitoxin degradation and release of CcdB toxin from the CcdA-CcdB complex. Free CcdB binds to its target, DNA gyrase, which induces the RecA-mediated SOS response and in turn activates other TA systems, finally leading to formation of persister cells [123].

### 5.3. TA-Induced Persistence in Bacterial Species Other than E. coli

The experimental data regarding persistence in bacterial species other than *E. coli* are restricted to a few examples. Overproduction of the native HicA toxin results in growth arrest and increases the number of persisters insensitive to ciprofloxacin or ceftazidime in *Burkholderia pseudomallei*, the causative agent of melioidosis. The persistence frequency is modulated depending on the level of HicA production. Moreover, deleting the *hicAB* locus from *B. pseudomallei* significantly decreased persister cell formation after exposure to ciprofloxacin, but not to ceftazidime [124]. Similarly, inactivation of *relE* toxin family genes influenced the frequency of persister cell formation in *Mycobacterium tuberculosis* as outlined further below [125]. Moreover, overproduction of the VapC type II toxin led to the production of dormant cells in *Mycobacterium smegmatis* [126]. The *shpAB* type II locus of *Salmonella* mediates persistence in a (p)ppGpp independent manner [127]. TA systems also have been shown to induce persister cell formation of Salmonella in macrophages during infection as described further below [128]. 

## 6. Persistence and Viable but Non-Culturable Bacterial States are Related

Viable but non-culturable (VBNC) bacteria are non-dividing cells that possess low metabolic activity in response to environmental stresses. VBNC cells generally do not grow on standard microbiological media but may be revivable under certain circumstances. Persister cells and VBNC cells are closely related. Both cell types are part of a “dormancy continuum” that may arise by related mechanisms, but which are found in different physiological positions on the dormancy range [129]. Since TA systems are implicated in mediating persistence, the relationship between VBNC cells and persisters was supported by showing that VBNC cells express higher levels of *hipBA* and *relBE* genes than growing cells [130]. Thus, TAs may play roles in the modulation of both persistence and the VBNC state [129].

## 7. Deciphering the Roles of TA Modules in Pathogenicity

### 7.1. Mycobacterium tuberculosis: TA Proteins, Infection and Dormancy

*M. tuberculosis* causes a serious and prolonged pulmonary infection that has a high mortality rate if untreated [131]. In addition to strategies that promote the evasion of host immune defenses, entry of a subpopulation of cells into a dormant state is a major factor in the protracted infection induced by *M. tuberculosis*. Intermittent reawakening of this reservoir of latent infection triggers the recurrent relapses that typify the disease [132]. The rise of antibiotic resistant strains of *M. tuberculosis*, which in certain cases are resistant to multiple drugs, adds another challenging layer of difficulty to tuberculosis treatment [133].

As described above, TA modules are stress response elements that dampen physiological activity by, for example, inhibiting protein translation. A potential outcome of this metabolic shutdown is entrance into a persistent state in which cells are tolerant of antibiotics [64,102]. Analogously, the dormant state in *M. tuberculosis* is characterized by low metabolic activity and consequent insensitivity to antibiotic treatment. There is emerging evidence that latency in *M. tuberculosis* and persistence may be related phenomena that are mediated in part by TA cassettes through their roles in modulating physiological activity [134,135]. Indeed TA proteins are enriched in nutrient-starved cultures of *M. tuberculosis*, which suggests a role for TAs in progression into dormancy [136]. Intriguingly, *M. tuberculosis* harbors the most putative TA genes (>80) on a single genome that have been catalogued to date among bacterial species, although the functions of many of these genes remain unclear [39,137]. Some of the encoded TA cassettes, including certain VapBC modules which are the most abundant TAs in *M. tuberculosis*, have been characterized genetically and biochemically and have provided general insights into TA activity. For example, numerous *M. tuberculosis* toxins are sequence-specific endoribonucleases that cleave mRNA and are counteracted by the cognate antitoxins [138,139,140,141,142,143,144,145,146,147,148,149,150,151]. In contrast, a VapC homologue instead was shown recently to inhibit translation by cutting specifically a subset of mycobacterial tRNAs [152]. Elucidation of the tertiary structure of a mycobacterial VapBC complex led to the design of custom peptides that interfere with formation of the complex [153]. Moreover, degradation of a HigA antitoxin of *M. tuberculosis* unexpectedly was inhibited by a chaperone protein, which may reflect a broader role for chaperones in regulating TA activity [154,155].

In addition to investigating TA activity and diversity in *M. tuberculosis*, numerous strands of evidence support a role for TAs in mycobacterial pathogenesis. The bacterium encodes two functional *relBE* cassettes and one *yefM-yoeB* module [39,133,156]. The RelE and YoeB toxins are homologues, whereas the RelB and YefM antitoxins are unrelated [157,158]. The mycobacterial *relBE* and *yefM-yoeB* operons are up-regulated in response to nitrogen starvation and oxidative stress, and down-regulated in response to hypoxia [133,137,159]. These conditions are relevant to those prevailing during *M. tuberculosis* infection. Increased expression of one of the numerous mycobacterial *vapBC* homologues as well as a *higBA* locus also occurs during hypoxia [137]. Similarly, enhanced expression of the *relE* and *yoeB* toxin genes is evident following exposure to certain antibiotics, which consequently promotes entry into a persistent state under laboratory conditions [125,143,160]. However, deletion of the homologues affected neither mycobacterial survival nor persistence in mice [125]. In contrast, although expression of the *relBE* and *yefM-yoeB* genes was not detectable in the early or middle stages of infection following phagocytosis of *M. tuberculosis* by human macrophages, expression of genes encoding one of the two RelE toxins, the YoeB toxin, and one of the two RelB antitoxins was apparent in the late infection stage [156]. As macrophages fulfill a vital function in innate immunity by phagocytosis of infecting microbes, the expression of TA genes specifically at the late infection stage implicates the genes in mycobacterial survival in this hostile environment.

*M. tuberculosis* encodes ten MazEF homologues [39,138,161]. The biochemical features of several of these complexes have been investigated which has revealed that the mycobacterial MazF toxins are sequence-specific endoribonucleases that cleave mRNA, rRNA or tRNA and which are counteracted by the cognate MazE antitoxins [40,138,140,142,145,150,162,163,164]. Individual MazF toxins are up-regulated in response to stresses that mimic those that occur during infection including nutrient depletion and hypoxia, as well as by antibiotic exposure and entry into a non-culturable state [136,160,165,166]. Persister cell formation by *M. tuberculosis* also is linked to *mazF* expression [125,135,148,161]. Moreover, expression of one of the *mazF* homologues is coordinated with that of a gene that encodes a factor implicated in progression of the mycobacterial cell cycle. Elevated expression of both genes occurs for prolonged periods in the lungs and spleens of infected mice suggesting that this MazF homologue is involved in adaptation to the distinctive conditions that *M. tuberculosis* encounters during colonization [167]. Intriguingly, three of the mycobacterial MazF toxins act cooperatively to provide protection during oxidative and antibiotic stresses, most likely by inhibiting metabolism and thereby inducing a persister-like state [161]. Simultaneous deletion of these *mazF* homologues in *M. tuberculosis* significantly reduced the bacterial load in infected guinea pigs compared to either the wild-type strain or strains deleted of single *mazF* or *relE* toxin genes. The concomitant reduced pathology by the triple deletion mutant strain was more pronounced in the spleen and the liver than in the lungs, which implies that MazF toxins may be involved in the spread of *M. tuberculosis* from the lungs to other organs [161].

Expression of certain *vapBC* loci in *M. tuberculosis* is activated during macrophage infection or under hypoxic conditions [137]. TA loci, most notably certain *vapBC* genes, also are up-regulated in drug-tolerant *M. tuberculosis* isolated from patients [168]. Nevertheless, although 50 of the known TA modules in *M. tuberculosis* are *vapBC* homologues [135], there is scant additional evidence that implicates these complexes directly in mycobacterial pathogenesis. The numerous *vapBC* genes may possess redundant or overlapping functions, which render it difficult to pinpoint a role in infection. Analogously, *M. tuberculosis* harbors three *higBA* loci but, apart from up-regulation in antibiotic-tolerant persister cells [135], there is no established role for these homologues in mycobacterial virulence.

### 7.2. TAs in Virulent Enterobacteriaceae: Escherichia coli and Salmonella typhimurium

TA genes were first described on plasmids resident in *E. coli* [169,170]. Much of the intervening research on elucidating the functional, biochemical and structural features of TA complexes has centered on the well-characterized *E. coli* K-12 laboratory strain [6,11,50,64,171,172,173,174,175,176,177,178,179,180] (Figure 2). However, *E. coli* species are very diverse genetically and encompass both commensal and pathogenic strains. The latter may cause intestinal and extraintestinal infections of varying severity. Uropathogenic *E. coli* (UPEC) are a sub-category of the extraintestinal group and are responsible for the majority of urinary tract infections (UTI) in humans [181]. Individual deletions of the seven type II TA loci from a UPEC strain revealed that only mutants lacking the *pasTI* (also known as *yfjGF*) genes were outcompeted by the wild type strain in the kidneys, but not in the bladder, in a mouse UTI model system. The *pasTI* deletion also reduced kidney colonization under non-competitive conditions. The deletion strain colonized the mouse gastrointestinal tract normally [182]. Thus, the *pasTI* genes may play a crucial role in UPEC migration from the lower to upper urinary tract. The mechanism by which the *pasTI* locus mediates kidney colonization is unknown. However, as the genes are vital for persister cell formation by the UPEC strain, but not the K-12 strain, under laboratory conditions and also participate in stress resistance of UPEC strains *in vitro*, the complex may help UPEC strains to overcome defensive host factors including amino acid limitation and oxidative and nitrosative stresses [182]. Binding of the PasT toxin (also known as RatA) to the 50S subunit of the bacterial ribosome blocks its interaction with the 30S subunit. PasT-mediated inhibition of the formation of 70S ribosomes thus prevents translation initiation [183]. It remains to be resolved how this activity integrates with kidney colonization by UPEC strains, but down-regulation of protein synthesis may be highly advantageous under specific stress conditions that these strains encounter within certain host niches in the urinary tract.

In contrast with *pasTI*, deletion of either the *yefM-yoeB* or *hha-tomB* (also known as *hha-ybaJ*) TA loci in a UPEC strain reduced competitiveness in the bladder, but not in the kidneys, in a mouse UTI model [182]. As noted above, transcriptional repression of certain rare codon tRNAs by the Hha toxin inhibits the production of surface fimbriae that are essential for biofilm formation by pathogenic *E. coli* [75]. The TomB protein blocks the activity of Hha (Figure 3). Transcriptional repression of rare codon tRNAs by Hha also induces production of prophage lysis genes as well as the ClpP/ClpX proteases that liberate the toxin components of other TA complexes by antitoxin degradation. Cell lysis and biofilm disruption ensue which may explain the impaired competitiveness of the UPEC strain in bladder colonization [75].

*S. typhimurium* is a major foodborne pathogen. The chromosome of *S. typhimurium* possesses at least six putative or established type I modules and 17 type II TA modules [128,184]. Individual deletions of type II TA loci modestly reduced replication of *S. typhimurium* in bone marrow-derived macrophages and decreased persister cell formation [128]. In a separate study, certain type I and type II TAs were shown also to differentially modulate *S. typhimurium* survival in fibroblast and epithelial cell lines [184]. The type II TAs in *S. typhimurium* include a functional *higBA* locus denoted *sehAB* [185]. Transcription of *sehAB* increases when *S. typhimurium* is cultivated under laboratory conditions that are known to activate genes involved in intracellular proliferation. Although the genes also are up-regulated in mouse macrophages that are infected with *S. typhimurium*, the *sehAB* locus is not required for bacterial replication either in this cell line, in bone marrow-derived macrophages, or in HeLa cells [185]. *S. typhimurium* causes infection following oral ingestion of contaminated food. Deletion of the *sehAB* genes reduced the virulence of *S. typhimurium* administered by this natural route in mice, but had no effect when the mutant was inoculated intraperitoneally. Nevertheless, once an infection was established by the oral route, the deletion strain survived as effectively as the wild-type strain. Thus, the *sehAB* genes are implicated in the initial stages of infection but not in systemic spread of *S. typhimurium*. Moreover, the SehAB complex fulfills a particularly vital role in maintaining the bacterium within mesenteric lymph nodes that are located in the membrane which attaches the intestine to the abdominal wall and which are crucial for preventing the extraintestinal spread of infections [185].

The SehA toxin is a homologue of HigB which is a ribosome-dependent endoribonuclease [186]. SehA similarly may act as a virulence factor in *S. typhimurium* by inhibiting protein translation in response to signals found specifically in mesenteric lymph nodes and other tissue niches. This inhibition may diminish metabolic activity transiently in *S. typhimurium* which, by an unknown mechanism but that potentially involves a persister-like state, may promote intracellular survival [185]. Deletion of a locus that encodes a RelE-like toxin [127] impaired competitiveness with wild-type *S. typhimurium*. The mutant strain also formed fewer persister cells in mesenteric lymph nodes of mice infected by the oral route [128]. Although the number of studies that have been undertaken are as yet limited, it is apparent that TAs contribute to the pathogenicity of *S. typhimurium* and may prove to be important virulence determinants in the establishment and progression of infection.

### 7.3. TAs in Other Gram-Negative Pathogens

In addition to *E. coli* and *S. typhimurium*, a handful of studies have described potential roles for TA complexes in the virulence of other Gram-negative pathogens. *Leptospira* spp. are causative agents of the most globally widespread zoonotic infectious disease. Leptospirosis is especially prevalent in tropical climates and in its most severe form can impair multiple organs, notably the kidneys, liver, meninges and brain [187]. Expression of the *chpBK* and *mazEF* TA genes was elevated in human macrophages infected with *Leptospira interrogans* compared to cultures grown in laboratory medium. The wild-type strain and mutants deleted of either module induced early-stage macrophage apoptosis to similar levels. However, the mutants induced late-stage apoptosis less effectively than wild-type suggesting that the *chpBK* and *mazEF* TA loci have roles in cellular necrosis by *L. interrogans* [188].

*Vibrio cholerae* is the etiologic agent of cholera, a potentially life-threatening, acute diarrheal disease with epidemic potential [189]. The superintegron (SI) of *V. cholerae* is a genomic region with high genetic flexibility that is thought to allow a rapid response to environmental changes and niche adaptation. The SI harbors an unusually high concentration of type II TA modules: 17 cassettes are dispersed within only a ~125-kb sequence. These TA loci are functional and may act in concert to ensure that the SI is maintained in *V. cholerae* by a similar mechanism of post-segregational cell killing as established for plasmid-located TA genes [190]. Separate deletions of two *relBE* modules within the SI impaired colonization by the mutants of the mouse intestine in competition with the wild-type strain. Deletions of the other five *relBE* homologues had no discernible effect on virulence [86]. Deletion of the *relBE* loci implicated in colonization also modestly perturbed biofilm formation by *V. cholerae*, although no effects on antibiotic resistance, the oxidative stress response or sensitivity to bile salts were observed in any of the mutants [86].

*Haemophilus influenzae* is a commensal resident of the normal airway microflora, but also is an opportunistic pathogen that causes a range of respiratory tract infections, including otitis media in children, as well as more systemic disease, including bacteraemia and meningitis [191]. *H. influenzae* strains are classified as encapsulated or unencapsulated based on the presence or absence, respectively, of a protective polysaccharide capsule that surrounds the cell. Unencapsulated strains also are termed nontypeable *H. influenzae*. Nontypeable *H. influenzae* strains possess at least four *vap* TA loci. The effects of the separate and simultaneous deletion of two of these loci were assessed in primary human respiratory epithelial tissue, which serves as an effective *in vitro* model for the upper airway [192]. Survival of the single and double deletion *vap* mutants in this model was attenuated in comparison with the wild-type strain. Similarly, the mutant strains persevered significantly less well and induced less middle ear inflammation than wild-type nontypeable *H. influenzae* in a chinchilla model of otitis media [192]. Analogous results were obtained with deletion of a *higBA* locus [193]. Thus, *vap* and *higBA* modules enhance the pathogenicity and survival of nontypeable *H. influenzae* in both *in vitro* and *in vivo* infection models.

Finally, Rickettsia are obligate intracellular bacteria that are transmissible from arthropods to humans in whom several *Rickettsia* spp. can induce serious, sometimes fatal, infections [194]. *Rickettsia* spp. with multiple type II TA modules promote higher levels of host cell apoptosis than a species with fewer TA genes. A VapC toxin homologue may be implicated in this effect [195,196,197].

### 7.4. TAs and Virulence in Gram-Positive ESKAPE Pathogens

The ESKAPE group of pathogens is responsible for a majority of hospital-acquired, multiresistant infections in the USA [198]. Among this group are *Staphylococcus aureus* and *Enterococcus* spp. which are Gram-positive, commensal bacteria that colonize the skin and respiratory tract, and the gastrointestinal tract, respectively. However, both species also can cause disease that ranges from transient infections to more serious, life-threatening illnesses. The recent emergence of antibiotic multiresistant strains, especially in hospital environments, has made treatment of staphylococcal and enterococcal infections much more problematic. Although TA loci are widespread in *S. aureus* [199], there are no definitive reports that link these TAs with pathogenicity. Nevertheless, TAs are implicated in staphylococcal persistence [200] and may regulate the production of important virulence factors in *S. aureus* [201,202]. Moreover, the MazEF complex influences the sensitivity of *S. aureus* to β-lactam antibiotics [203].

TA genes are ubiquitous in enterococci, including on transferable resistance plasmids [27,157,204,205]. Virulence plasmid pAD1 encodes a type I TA complex (*par*) in which translation of a 33 amino acid toxin specified by RNA I is blocked by a 66 nucleotide regulatory antitoxin, RNA II. Homologues of *par* are widespread on bacterial genomes [206]. Deletion of the gene for a chromosomal RNA II in *Enterococcus faecalis* enhanced virulence both in an insect infection model and in a mouse UTI model. The mutant strain also survived better than the wild-type strain in mouse macrophages, was more acid resistant, and showed improved growth when exposed to oxidative stress or bile salts [207]. The expression of RNA II antitoxin was downregulated in response to oxidative stress or bile salts suggesting that modulating expression of this chromosomal *par* locus is key to survival under the hostile conditions encountered by *E. faecalis* during infection. Moreover, the strain deleted of the RNA II gene showed global proteome changes, including elevated expression of an important transcription factor that is involved in virulence and the oxidative stress response [207]. These alterations correlate with the adaptation of *E. faecalis* from the free-living to colonizing state. Despite its role in regulating translation of the cognate RNA I toxin gene, deletion of the RNA II antitoxin gene did not detectably boost expression of RNA I [207]. Thus, the molecular mechanisms that underpin the enhanced virulence of the *E. faecalis* antitoxin mutant require further investigation. The potential roles of TA modules in the virulence capacity of other Gram-positive bacteria, such as *Streptococcus* spp. [208] and *Clostridium* spp. [209], which encompass a number of major human pathogens, largely is *terra incognita*.

## 8. Conclusions

Recent studies have highlighted key roles for a range of TA modules in the virulence potential of numerous important human pathogens. The emerging picture is that toxin activation in these complexes is crucial for modulating bacterial metabolism to promote pathogen survival in the harsh conditions faced during infection. The development of a persistent or semi-dormant state that is mediated at least in part by TA complexes appears especially important. The presence of genes for multiple TAs on pathogen genomes [33] complicates dissection of these loci, which may have redundant or overlapping functions. Cryptic loci, especially those for type I modules that are genetically compact [12], may obscure further the contribution of TAs to virulence. Nevertheless, the systematic single and multiple deletions of TA modules from pathogen genomes followed by testing in relevant virulence models is a potent strategy to investigate the contribution of TAs to disease. Moreover, TA modules implicated in virulence are prospective targets for novel prophylaxis and therapy approaches that are urgently required in an era of expanding antibiotic resistance.
